# Role of gut microbiota in the development of insulin resistance and the mechanism underlying polycystic ovary syndrome: a review

**DOI:** 10.1186/s13048-020-00670-3

**Published:** 2020-06-17

**Authors:** Fang-fang He, Yu-mei Li

**Affiliations:** Department of Assisted Reproduction, Xiangya Hospital affiliated Central South University, Changsha, 410008 People’s Republic of China

**Keywords:** PCOS, Gut microbiota, Insulin resistance, Hyperandrogenism

## Abstract

Polycystic ovary syndrome (PCOS) is a complex endocrine and metabolic disorder. Typically, it is characterized by hirsutism, hyperandrogenism, ovulatory dysfunction, menstrual disorders and infertility. To date, its pathogenesis remains unclear. However, insulin resistance (IR) is considered as the primary pathological basis for its reproductive dysfunction. On the other hand, a condition in which insulin is over-secreted is called hyperinsulinemia. IR/Hyperinsulinemia is associated with chronic inflammation, hormonal changes, follicular dysplasia, endometrial receptivity changes, and abortion or infertility. Additionally, it increases incidence of complications during pregnancy and has been associated with anxiety, depression, and other psychological disorders. Gut microbiota, the “second genome” acquired by the human body, can promote metabolism, immune response through interaction with the external environment. Gut microbiota dysbiosis can cause IR, which is closely linked to the occurrence of PCOS. This article reviewed recent findings on the roles of gut microbiota in the development of insulin resistance and the mechanism underlying polycystic ovary syndrome.

## Introduction

Polycystic ovary syndrome (PCOS) is one of the most common gynecologic endocrine disorders, widely considered as a top cause of infertility [1, 2]. Globally, it affects 8–13% of women in the reproductive age bracket [3, 4]. Besides, PCOS has been linked to a higher risk of metabolic disorders, including insulin resistance (IR), glucose intolerance, type 2 diabetes mellitus(T2DM), dyslipidemia, and cardiovascular diseases [5, 6]. Therefore, there has been an increasing focus on the effect of PCOS on women’s health in general [7]. Till now, there is an underlying uncertainty on etiology and pathogenesis of PCOS. However, studies have outlined its multifactorial origin, which involves genetic factors, neuroendocrine, lifestyle, immune, and metabolic dysfunctions [8]. Precisely, IR is one of the primary pathologic changes leading to the occurrence and development of PCOS [5, 9–11]. Additionally, other studies have suggested that appearance of IR could be caused by gut microbiota dysbiosis [12–14].

Gut microbiota is the most plentiful and functionally critical microflora which encompasses about 10^14^ resident microorganisms and commensals within human intestinal tract [15]. Studies have closely linked the gut microbiome with level of human metabolism [16]. Actually, the occurrence and development of a variety of endocrine and metabolic diseases is affected by dynamics of structure in the intestinal flora. Tremellen et al. proposed a hypothesis called DOGMA (dysbiosis of gut microbiota), which explains a possible sequence of events in the pathogenesis of PCOS as follows: 1) Obesity or high sugar, high-fat and low dietary fiber diet causing imbalance on intestinal flora, thus destroying connection between intestinal epithelial cells, which increases permeability of the gut mucosal; 2) Leaky gut may perhaps cause leakage of lipopolysaccharide (LPS) into systemic circulation, and the resultant activation of the immune system might interfere with functioning of insulin receptor, causing IR; 3) IR/Hyperinsulinemia might promote synthesis of testosterone, hence meddling with follicular development [17]. Actually, gut microbiota composition in patients with PCOS was significantly altered compared with women without PCOS as shown in recent study. Moreover, intestinal flora disorders have been closely associated with clinical symptoms such as obesity and IR [18–22]. However, there lacks full understanding on mechanism of gut microbiota in patients with PCOS [23, 24]. In this mini-review, we discuss recent evidences as follows: 1) the role of IR in the development of PCOS; 2) correlation between gut microbiota and PCOS;3) potential mechanism underlying association between IR and gut microbiota in patients with PCOS 4) possible ways of overcoming gut microbiota dysbiosis with the aim of improving treatment for patients with PCOS.

## Overview of the relationship between IR and PCOS

Insulin resistance (IR) is one of the manifestations of polycystic ovary syndrome (PCOS). It causes ovulatory dysfunction and development of endometrial disorder, of which both of them lead to infertility. Also, IR has long-term and detrimental effects on metabolism of patients with PCOS. Studies have proposed IR as one of the most common endocrine characteristics in patients with PCOS; however, this condition has not been incorporated in diagnostic criteria for PCOS. Regardless of obesity, 50% of patients with PCOS have IR [8, 25–27].

### IR and the development of follicle in patients with PCOS

Anovulation or oligovulation is a common symptom of PCOS. IR and its compensatory hyperinsulinemia are intrinsic factors in the development of PCOS and play a key role in producing the excess androgen in PCOS [5, 28–30]. The excess insulin production can trigger insulin receptor of pituitary gland, to release luteinizing hormone (LH) [31], and promote secretion of androgen by ovary and adrenal gland; Besides, it can inhibit the synthesis of hepatic sex binding globulin (SHBG), and increase the levels of free testosterone (T) [32]. Excess androgen secretion may lead to hirsutism, acne, alopecia symptoms, and may inhibit the growth and development of ovarian follicles. It is the most common reason of barren secondary to ovulatory dysfunction [33]. Moreover, insulin can directly regulate the development of the ovarian follicle and hormone secretion through the insulin receptors in the follicle membrane cells. Also, insulin can enhance activity of insulin-like growth factor-1 (IGF-1) receptor in the ovary, which increase its levels of free IGF, promoting e production of androgen [34].

### IR and the endometrial receptivity in PCOS

Changes in endometrial function may also be a significant cause of low fertility in PCOS patients. Insulin resistance, as a characteristic metabolic feature in PCOS, affects the physiological function in uterine endometrium relating to endometrial receptivity(ER) [35, 36]. The ER of patients with PCOS is worse than that of normal women [37]. The condition can lead to a reduction in the levels of glucose transporter 4(GLUT-4) and other glucose transporter proteins in the late stage of endometrial hyperplasia, leading to the shortage of glucose supply in endometrial cells thus the onset of endometrial development disorder [9, 38, 39]. Moreover, the hyperandrogenism induced by IR can inhibit the growth and activity of endometrial cells by interfering with glucose metabolism, thus inhibiting decidual differentiation [40]. Therefore, under IR/hyperinsulinemia, poor ER may be aggravated in PCOS [41].

### IR and the metabolic abnormality in women with PCOS

IR is closely associated with glucose and lipid metabolism disorders, amino acid metabolism, and other adverse consequences. Dyslipidemia is the most common metabolic disorder in PCOS patients [42]. It has a prevalence rate of 70% and is positively correlated with IR [43]. IR reduces ability of the insulin to inhibit lipase, leading to a high level of free fatty acids and an increase in the risk of obesity and cardiovascular diseases in PCOS patients. Subsequently, hyperinsulinemia inhibits lipolysis through fibrinolysis, thereby inducing arterial hypertension [44]. It is reported that patients with PCOS are 5 to 7 times more likely to develop T2DM than patients without PCOS because of abnormal glucose metabolism caused by IR or hyperinsulinemia [45].

## Gut microbiome contributes to insulin resistance

Several animal and human experiments have confirmed that IR is closely interconnected to the nature of gut microbiome [12–14]. As early as 2004, Gordon et al. transplanted a healthy intestinal flora of mice into germ-free mice. After feeding, there was an increase in body fat of the germ-free mice, and insulin resistance was witnessed. This was the first finding to associate intestinal flora with insulin resistance [46]. Elsewhere, variations were indentified in the gut microbiome between T2DM, impaired glucose tolerance, and the control group of patients using macrogenomic sequencing [47]. Also, Vrieze et al. transplanted the gut microbiome of healthy people into patients with metabolic syndrome; and after six weeks, an increase in insulin sensitivity of the recipients was observed [48]. Several studies have designated that gut microbiota plays a significant role in the development of obesity, obesity-associated inflammation, and insulin resistance. Notably, for the first time, Cani et al. proposed that “endotoxemia” which is producedby gut microbiome could be an essential factor in initiing inflammatory activities leading to obesity and insulin resistance [49]. Usually, gut microbiome promotes energy absorption by enhancing the synthesis of triacylglycerol, and inhibiting the oxidation of fatty acids, and this may affect the energy balance of the human body, leading to IR. Dysbiosis of gut microbiota can increase intestinal mucosal permeability. Actually, a variety of inflammatory mediators such as lipopolysaccharide (LPS), and the branch-chain amino acid (BCAA) are produced by intestinal microflora. The BACC activates the immune response of the body, whereas the inflammatory mediators activate toll-like receptors 4 (TLR4), resulting in reduced sensitivity to insulin.

## PCOS and gut microbiota

Currently, there is a preference for researchers to use sequencing technology to classify, identify, and accurately quantify intestinal bacteria. Lately, several leading studies have demonstrated that microbiota dysbiosis occurs in PCOS animal models and PCOS patients [18–22]. Of note, a change in the overall composition of the gut bacteria has been associated with PCOS, including a decrease in alpha and beta diversity. Moreover, changes in the relative abundances of specific types of gut bacteria have been associated with clinical manifestations of PCOS(Table 1).

**Table 1:** Summary of major microbiome studies involving PCOS

Study Author/year	Country	diagnostic criteria	Sample groups	Sequencing	diversity change	Microbial composition change
**Kelley 2016** [50]	**USA**	**NA**	**Letrozole-induced PCOS mouse model/placebo**	**16S rDNA sequencing** **V4**	**↓α diversity** **β diversity:** **PCoA of the UniFrac distances: a significant shift**	**↑Lachnospiraceae, Erysipelotrichaceae, Ruminococcacea** **↑Allobaculum, Blautia, and Ruminococcus** **↓S24–7 and 1 in the family Rikenellaceae, genus Alistipes**
**Guo Y 2016** [7]	**China**	**NA**	**Letrozole-treated rats (*n*** **= 24)/control (n = 8)**	**16S rDNA sequencing** **V3**	**NA**	**↓Lactobacillus, Ruminococcus, and Clostridium** **↑Prevotella**
**Lindheim 2017** [18]	**Austria**	**Rotterdam Criteria**	**PCOS (n = 24)/control (*n*** **= 19)**	**16S rDNA sequencing** **V1-V2**	**↓α diversity** **β diversity:** **Unweighted UniFrac analysis: significant clustering**	**↓Phylum Tenericutes, the order ML615J-28 (belonging to the phylum Tenericutes)** **↓Family S24–7 (belonging to the phylum Bacteroidetes)**
**Liu 2017** [18]	**China**	**Rotterdam Criteria**	**PCOS (non obese PCOS,*n*** **= 12; obese PCOS,*n*** **= 21)/controls (*n*** **= 9 non obese control;*n*** **= 6 obese control)**	**16S rDNA sequencing V3–4**	**↓α diversity** **β diversity:** **CO group was more similar with that of PN and PO groups, rather than CN group**	**↑LPS-producing bacteria** **↓Spore forming species** **↓Akkermensia (of the phylum Verrucomicrobia) and Alistipes, Corprococcus, Ruminococcus**
**Torres 2018** [20]	**Poland**	**Rotterdam Criteria**	**Women with PCOM ** (***n*** **= 42)/control healthy women** **(*n*** **= 48)/women with PCOS (*n*** **= 73)**	**16S rDNA sequencing V4**	**↓α diversity in women with PCOS compared with healthy women** **β diversity was correlated with hyperandrogenism**	**↑Porphyromonas spp., Bacteroides coprophilus, Blautia spp., Faecalibacterium prausnitzii in women with PCOS compared with healthy women**
**Insenser 2018** [21]	**Spain**	**Rotterdam Criteria**	**PCOS (n = 15)/16 non hyperandrogenic control women (*n*** **= 16) and control men (*n*** **= 15)**	**16S rDNA sequencing V4**	**↓α diversity in women compared with men** **↓β diversity in obese patients with PCOS compared with their lean counterparts**	**↑Catenibacterium, Kandleria genera**
**Zeng 2018** [22]	**China**	**Rotterdam Criteria**	**IR-PCOS (n = 9, PCOS with insulin resistance),** **NIR-PCOS (n = 8, PCOS alone), and healthy controls (*n*** **= 8)**	**16S rDNA sequencing V3–4**	**↓α diversity in IR-PCOS compared to healthy controls** **β diversity(weighted UniFrac and PCoA analysis):samples from the HC group can be clearly discriminated from the IR-PCOS group**	**↑Prevotellaceae in IR-PCOS and NIR-PCOS compared to healthy controls** **↑Bacteroides in IR-PCOS and NIR-PCOS compared to healthy controls**
**Sheman** **2018** [84]	**USA**	**NA**	**Model of prenatal androgen exposure rats/maternal hyperandrogenemia by single-injection of testosterone cypionate or sesame oil vehicle**	**16S rDNA sequencing**	**↑α diversity in PNA animals** **β diversity:** **the PNA animal samples were further apart.**	**↑Nocardiaceae and Clostridiaceae** **↓Akkermansia, Bacteroides, Lactobacillus, and Clostridium**
**Zhang 2019** [52]	**China**	**NA**	**PCOS (*n*** **= 38)/control (*n*** **= 26)**	**16S rDNA sequencing V3-V4, deep shotgun metagenomic sequencing**	**β diversity:** **weighted and unweighted UniFrac analysis:** **significant clustering**	**↓Faecalibacterium, Bifidobacterium, and Blautia** **↑Parabacteroides, Bacteroides, Lactobacillus, Oscillibacter, Escherichia/ Shigella, and Clostridium**
**Chen 2019** [98]	**China**	**Chinese Criteria** **2012**	**non⁃acne PCOS patients(*n*** **= 10), MSA⁃PCOS,n = 10)/ healthy controls (n = 10)**	**16S rDNA sequencing** **V3-V4**	**↓α diversity** **β diversity:** **weighted UniFrac analysis:samples from the HC group can be clearly discriminated from PCOS**	**↑Bacteroides** **↓Prevotella, Faecalibacteruim**
**Zhou 2020** [99]	**China**	**Rotterdam Criteria**	**obese PCOS (OG,*n*** **= 30), non-obese PCOS (NG,N = 30) /healthy women (NC,n = 30) healthy but obese women (OC,*n*** **= 11)**	**16S rDNA sequencing** **V3-V4**	**α diversity: Simpson index of OTU**: **NG was significantly higher than that in NC** **β diversity: No significant difference**	**↑OG:Lactococcus, Paraprevotella, Alloprevotella** **↑NG:Coprococcus_2, Lactobacillus, Prevotella_7**
**Qi 2019** [53]		**NA**	**PCOS (*n*** **= 50)/control (n=43)**	**Whole-genome shotgun sequencing**	**α diversity:no significant** **difference**	**↑Bacteroides vulgatus**

### PCOS and microbiota diversity

Alpha(α) diversity has been suggested to correlate with the health of an ecosystem, and it can be used to estimate the abundance and diversity of species in an environmental community. Beta (β) diversity is known as the variety of the ecological environment (between habitat diversity). Several studies have reported a decrease in α diversity and a change in β diversity in patients with PCOS [18, 20]. Zeng et al. Zeng et al. found a lower number of observed operational taxonomic units (OTUs) and a lower Shannon index in IR-PCOS group of patients after comparing its structural and functional profiles of the gut microbial community withNIR-PCOS [22]. They further showed that hierarchical clustering could distinguish samples from the healthy control (HC) group with the IR-PCOS group [22]. Lastly, changes in α and β diversities may lead to changes in intestinal function, concerning insulin levels, glucose tolerance levels, and androgen levels, which may worsen the symptoms of PCOS.

### PCOS and microbiota composition

Studies have found a relationship between PCOS and the abundance of some gut microbiota at phylum, family, and genus levels. Many species of gut bacteria exist, 90% of which are composed of *Firmicutes* and *Bacteroides*, followed by *Actinomycete*, *Proteobacteria*, and *Clostridium*. In the phylum level, Kelley et al. were the first to confirm changes in intestinal flora in letrozole-induced PCOS model in mice, including a significant decrease in the total intestinal microbial species count and phylogenetic richness [50]. And this mainly manifested by a reduction in *Bacteroides* and an increase in *Firmicutes*. The rise in *Firmicutes* is closely caused by the occurrence and development of obesity, T2DM, and metabolic syndrome(MetS) [46]. Lindheim et al. observed a decrease in bacteria from*Tenericutes*(order ML615J) and *Bacteroidetes*(Family S24–7) phyla with a relative abundance> 1% [18], although there were no significant differences in the lower taxa [51]. At the family level, *lactobacilli* and *bifidobacteria* are beneficial bacteria that function by enhancing immunity and nutrient absorption. These bacteria were significantly reduced in PCOS patients. Zeng et al. observed a dramatic decrease in the amount of *Prevotellaceae* in PCOS patients compared to healthy counterparts. However, the level of *Prevotellaceae* was increased in similar models in rats, which suggested that the increase in *Prevotellaceae* might have induced an inflammatory response in the host. At the genus level, Torres et al. identified four taxa(*Anaerococcus, Odoribacter, Roseburia, and Ruminococcus*) that had a lower abundance in PCOS patients [20]. Similarly, Zhang et al. revealed that the abundance of *Faecalibacterium* (specifically *Faecalibacterium Prausnitzii*), *Bifidobacterium*, and *Blautia* were lower in PCOS patient [52]. The reduction of these bacteria might lead to changes in production of short-chain free fatty acids (SCFAs), which might affect the integrity of the intestinal barrier. In fact, gram-negative bacteria are known to produce lipopolysaccharides (LPS), which may induce inflammation, insulin resistance, and obesity by leaking into the blood-stream [49]. Notably, Liu et al. observed that some gram-negative bacteria belonging to the genera *Bacteroides* and *Escherichia/Shigella* significantly increased in the gut of PCOS patients [19]. Qi et al. reported *Bacteroides vulgatus* was significantly elevated in the gut microbiota in PCOS patients, accompanied by a reduction in the levels of glycodeoxycholic and tauroursodeoxycholic acids [53]. This report indicated that bile acid metabolism was one of the critical metabolic pathways affected by the gut microbiota changes in PCOS patients. Intestinal flora metabolism has been closely associated with the occurrence and development of diseases. The metabolic functions of intestinal microflora include the production of vitamins, short-chain free fatty acids (SCFAs) and conjugated linoleic acids, amino acid synthesis, bioconversion of bile acids, fermentation and hydrolysis of non-digestible foods, ammonia synthesis and detoxification [54]. Its mechanism of in relation to PCOS can be explored further by monitoring its alteration and association with metabolic indicators.

## Potential mechanism underlying the association between IR and gut microbiota of PCOS

**Fig. 1:**
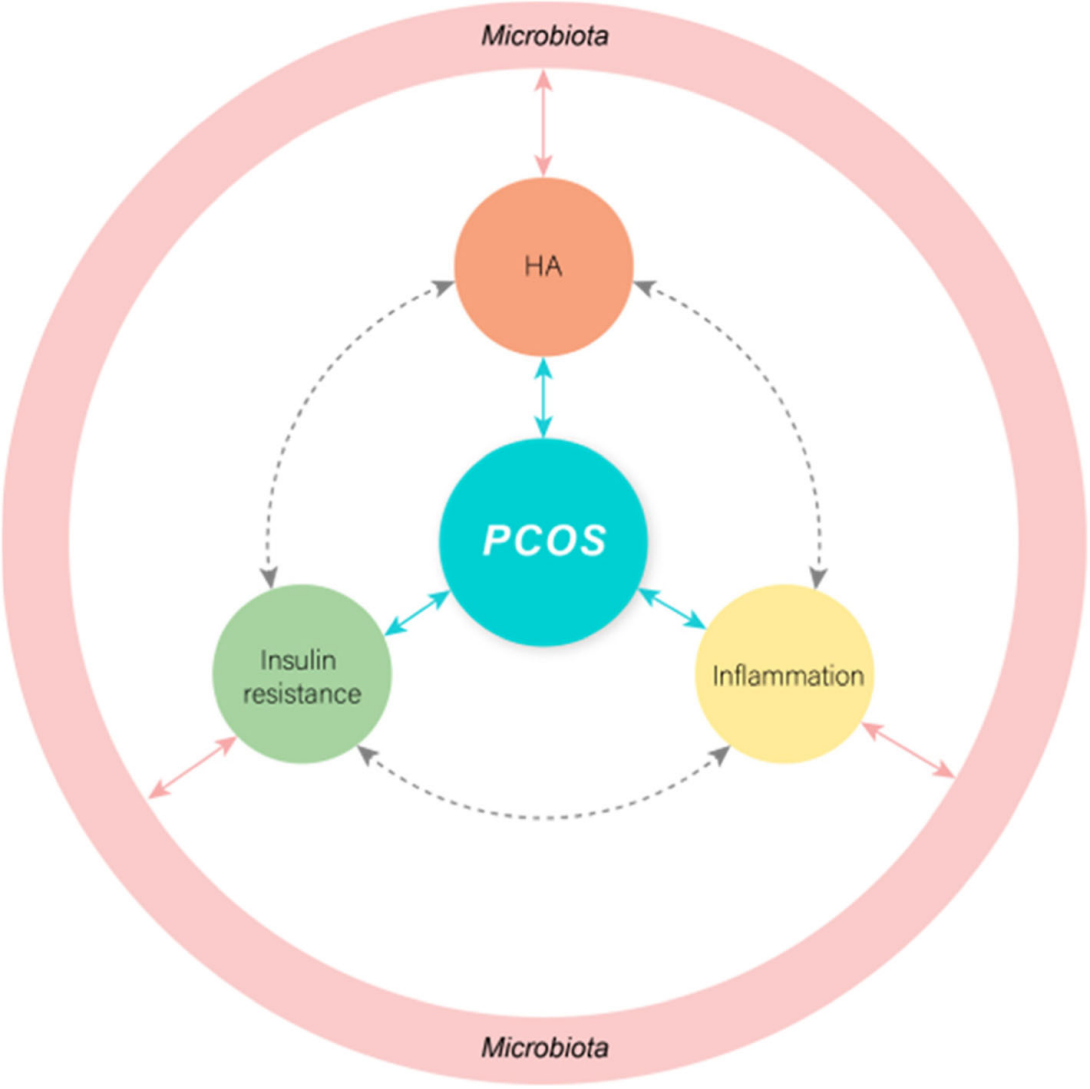
Crosstalk between the gut microbiota and the mammalian host in inflammation and metabolism. The gut microbiota can contribute to host insulin resistance, low-grade inflammation, and hyperandrogenism(HA) through a range of molecular interactions with the host and therefore can indirectly participate in the onset of PCOS

Currently, the pathogenesis of PCOS is still uncertain, and research on its etiology has maiorly focused on genetics, immunity, androgen exposure, and so forth. However, none of the above factors can fully explain the clinical manifestations of PCOS. On the other hand, intestinal flora is an indispensable “microbial organ” of the human body, which plays a critical role in sustaining human health. The signal pathway behind the insulin receptor has a crossover effect with the signal transduction of chronic subclinical inflammation [55, 56]. Studies have revealed that the occurrence of insulin resistance is associated with endotoxemia, chronic inflammatory response, short-chain fatty acids, and bile acid metabolism. Moreover, a significant imbalance in the intestinal flora has been observed in PCOS patients [57–60]. Given this, we speculated that gut microbiota might be involved in the pathogenesis of PCOS by mediating systemic low-grade inflammation and insulin resistance, affecting the changes in sex hormones, gut-brain axis, and other pathological mechanisms(Fig. 1).

### Endotoxemia

PCOS is a female reproductive endocrine disease closely related to chronic inflammation [28, 61]. Here, Tremellen et al. proposed the hypothesis of the “gut barrier-endotoxemia-inflammation mechanism”, which concerned the pathogenesis of PCOS [17]. Additionally, Lindheim et al. also observed less the diversity on intestinal flora for patients with PCOS patients. In this same study, changes in serum markers such as Zonulin, calprotectin, and LPS as a result of related to intestinal barrier damage and inflammation, such as Zonulin, calprotectin, LPS, were observed [18]. Actually, “endotoxemia” could play a role in the pathogenesis of PCOS by initiating inflammatory activity [17]. Notably, LPS produced by intestinal flora are vital molecules in the early development of inflammatory and metabolic diseases and have an endotoxin effect. Actually, after LPS is absorbed into the blood, LPS binding protein (LBP) binds to CD14 toll-like receptor complex (TRL-4) on the surface of innate immune cells, activates the downstream signaling pathway, the resulting immune system activation interferes with insulin receptor function, drives up serum insulin levels [62]. At the same time, the presence of a “leaky gut” would generate an increase in serum tumor necrosis factor-alpha (TNF-α) and interleukin6 (IL-6) which is mediated by endotoxin-induced activation of macrophages [63]. In PCOS patients, the expression of TNF and IL-6 is increased, which is related to IR [17, 64].Other studies also have shown that direct application of lipopolysaccharide to the blood circulation of mice and humans can increase fasting blood glucose and insulin levels [49, 65]. IR and chronic inflammation are closely related, which implies that when intestinal barrier function is impaired, endotoxin produced by intestinal flora enters the blood to cause chronic ovarian inflammation and IR, hence promoting the occurrence and development of PCOS.

### Short-chain free fatty acids (SCFAs)

Gut microbiome decomposes dietary fiber to produce three major SCFAs, which include acetate, propionate, and butyrate [66]. Butyrate, in particular, including acting as an energy source for intestinal epithelial cells; and anti-inflammatory effects [67]. The SCFAs also maintain the barrier function of the intestinal mucosa. Besides, they are important signal molecules that directly activate G-protein-coupled receptor 41 (GPR41) and GPR43 [68]. SCFA participates in glucose-stimulated insulin secretion from the pancreatic β-cells through interaction with the GPR41 and GPR43 receptors, improve insulin sensitivity and release of peptide hormones which control appetite [69, 70]. *Firmicutes* mainly produce butyrate, whereas *Bacteroides* produces acetate. On the high-fat diet in mice, supplementation of SCFA butyrate prevented development of insulin resistance and obesity by increasing energy expenditure [71]. In the recent study on PCOS and intestinal bacteria, lower abundance of several kinds of gut microbiome in women with PCOS are all known to synthesize SCFAs [20]. After four weeks of probiotics intervention in patients with PCOS, the abundance of *Lactobacillus* increased significantly, it can generate lactic acid to promote the growth of *Faecalibacterium,* which produces butyric acid, Promote the secretion of insulin [72].. These findings suggest that SCFAs protect intestinal barrier integrity and act on beta cells to promote insulin secretion, thus improving metabolism of PCOS [73].

### Bioconversion of bile acids

Bile acids (BAs) are signaling molecules that regulate glucose metabolism and promote insulin sensitivity mainly through its receptors: farnesoid X receptor (FXR) and G protein-coupled bile acid receptor 1 (GPBAR1). Besides assisting in fat absorption, the combination of BAs with receptors and G protein-coupled receptors (GGPCRs) on the cell surface activates signal transduction. Activation of nuclear receptor FXR improves the symptoms of hyperlipidemia and hyperglycemia in T2DM mice [74]. Sun et al. performed a metagenomic and metabolomic analysis of samples from patients who were newly diagnosed with T2DM and were treated with metformin for three days [60]. The analysis revealed a reduction in the abundance of *Bacteroides fragilis* and an increase in the bile acid, and glycoursodeoxy cholic acid (GUDCA) in the gut. Recent studies have found that altering bile acid metabolism may be valuable in the treatment of PCOS. Glycodeoxycholic acid induces intestinal group 3 innate lymphoid cell IL-22 secretion through GATA binding protein 3, whereas IL-22 in improves the PCOS phenotype [53]. These findings indicate that bile acids are involved in the regulation of IL-22 production, thus affecting ovarian function and insulin sensitivity in PCOS. As an internal environmental factor, intestinal microflora interacts with the host and may affect insulin secretion in several ways, thus participating in the occurrence and development of pathophysiological processes of PCOS.

### Amino acid synthesis

Branched-chain amino acids (BCAAs) was potentially harmful microbially microbial modulated metabolites. Recently, a recent study identified imidazole propionate as a microbially produced amino acid-derived metabolite and was shown to be present at higher concentrations in the portal and peripheral blood of patients with T2MD. Additionally, it worsens glucose tolerance in mice and impairs insulin signaling at the level of insulin receptor substrate (IRS) [75]. Pedersen et al. found Prevotella (in the human intestinal tract) species was involved in the synthesis of BCAA [58]. After its administration in mice fed with high fat, a significant increase in the BCAA content in the blood of the mice was observed after 2 weeks [76]. Subsequently, the mice showed varying degrees of IR after another three weeks. Zhang et al. found that leucine and valine levels in the follicular fluid of PCOS patients with IR were significantly increased. In the same study, higher levels of branched-chain amino acids increased the abortion rate and adverse pregnancy outcomes [77]. Prospective studies have also confirmed BCAA as a predictor of IR and diabetes [78]. The molecular mechanism of BCAA in pregnancy complications involves many signal transduction molecules, especially insulin receptor substrate 1 [79].The possible mechanism is that the metabolic disorder of amino acids might aggravate IR by changing glucose metabolism or inducing inflammation, leading to abortion in PCOS patients. Currently, there are few studies on the relationship between the gut microbiome and BCAA in PCOS patients, which calls for further investigation.

### Hyperandrogenism

IR directly or indirectly promotes the synthesis and secretion of androgen. Subsequently, hyperandrogenemia stimulates decomposition of visceral adipose tissue, leading to an increase in free fatty acids, which further aggravate the levels of IR. Such a sequence of events can ultimately form a vicious cycle between hyperandrogenemia and IR in PCOS, thus promoting the occurrence and development of PCOS. Barre et al. suggested that high intake of carbohydrate, hyperinsulinemia, hyperandrogenemia, and chronic low-grade inflammation are the four vital factors of pathophysiological changes in PCOS [80]. A metabolic dysfunction occuring predominantly in women diagnosed with hyperandrogenism and ovulatory dysfunction [81, 82]. In the pathogenesis of PCOS, insulin resistance and hyperinsulinemia could contribute to metabolic dysregulation by upregulating the production of ovarian androgen and increasing its bioactivity through decreased production of sex hormone-binding globulin. Excess androgen then leads to follicular development, maturation disorders, and follicular wall hyperplasia thickening which results resulting in ovulation disorders. Recently, a potential two-way interaction between sex hormones and intestinal flora has been proposed [83]. Prenatal androgen exposure can cause infant gut dysbiosis, hence altering the abundance of bacteria producing short-chain fatty acid metabolites. However, excess androgen in immature fetuses can result in long-term alterations of gut microbiota leading to an increased risk of developing PCOS [84]. Torres et al. discovered that treatment using letrozole adult mice was associated with a distinct shift in gut microbial diversity compared to its treatment in pubertal mice [85]. This finding showed that the timing of androgen exposure might influence metabolism dysregulation and the gut microbiome in PCOS, Which suggests that sex hormones can produce specific microbiota. Choi et al. also discovered that ovariectomized mice had fewer *Bacteroidetes* and more *Firmicutes* than normal mice. Similar to ovariectomy, analysis of the microbiome of castrated mice using quantitative PCR assay showed a reduction in both *Bacteroides* and *Ruminnococcaceae* compared with the control group [46, 86]. However, little is known about the mechanisms underlying sex steroid regulation of the gut microbiome. Further studies are needed to determine whether the changes in steroids in PCOS patients also affect the composition of intestinal microorganisms. And whether these changes are mediated by changes in glucose and lipid metabolism, cholesterol metabolism, and regulation of the host’s systemic or intestinal immunity.

### Gut-brain peptides

In recent years, studies have revealed that the pathological mechanism of PCOS is not confined to the dysfunction of the hypothalamic-pituitary-ovarian axis, but also involves the gut-brain axis. Besides, the hypothalamus is the centre of appetite regulation. The gut-brain axis plays a critical role in the regulation of appetite, food intake, glucose metabolism, energy maintenance, and body weight. Gastrointestinal hormones mainly include growth hormone-releasing peptide (Ghrelin), glucagon-like peptide-1 (GLP-1), cholecystokinin (CCK), and PYY. Gut microbiota and its metabolites cause insulin resistance and hyperinsulinemia by stimulating the secretion of gut-brain peptides and regulating inflammation pathway activation [24]. SCFAs stimulate the release of PYY and 5-HT in ileum and colon, wheres PYY inhibits gastrointestinal emptying and pancreatic secretion, slow intestinal peristalsis, and promote intestinal energy absorption [87, 88]. Lin et al. found an inverse correlation between PYY and insulin as well as between body mass index(BMI) and testosterone [89]. Compared to healthy women, patients with PCOS show lowered ghrelin levels, serotonin, PYY, which has a negative correlation with PCOS related parameters, such as waist circumference and testosterone levels [19]. *Bacteroidetes* mainly produce acetate, which is involved in cholesterol metabolism and lipogenesis. Also, *Bacteroidetes* inhibit the secretion of appetite-stimulating hormone [90]. Ghrelin is a hormone secreted by the gastric mucosa that stimulates appetite and is inhibited through ingestion [66, 90]. Although Houjeghani et al. found no difference in the ghrelin level between the PCOS group and the control group, many studies have found a negative correlation between serum ghrelin level and Homeostasis model assessment-insulin resistance (HOMA-IR) in PCOS patients [91]. Metformin usage in the treatment of PCOS has been associated with an increase in the levels of ghrelin, PYY, GLP-1, and GIP [92]. However, potential interactions of these treatments with the gut-brain axis in PCOS would be of much interest.

**Fig. 2:**
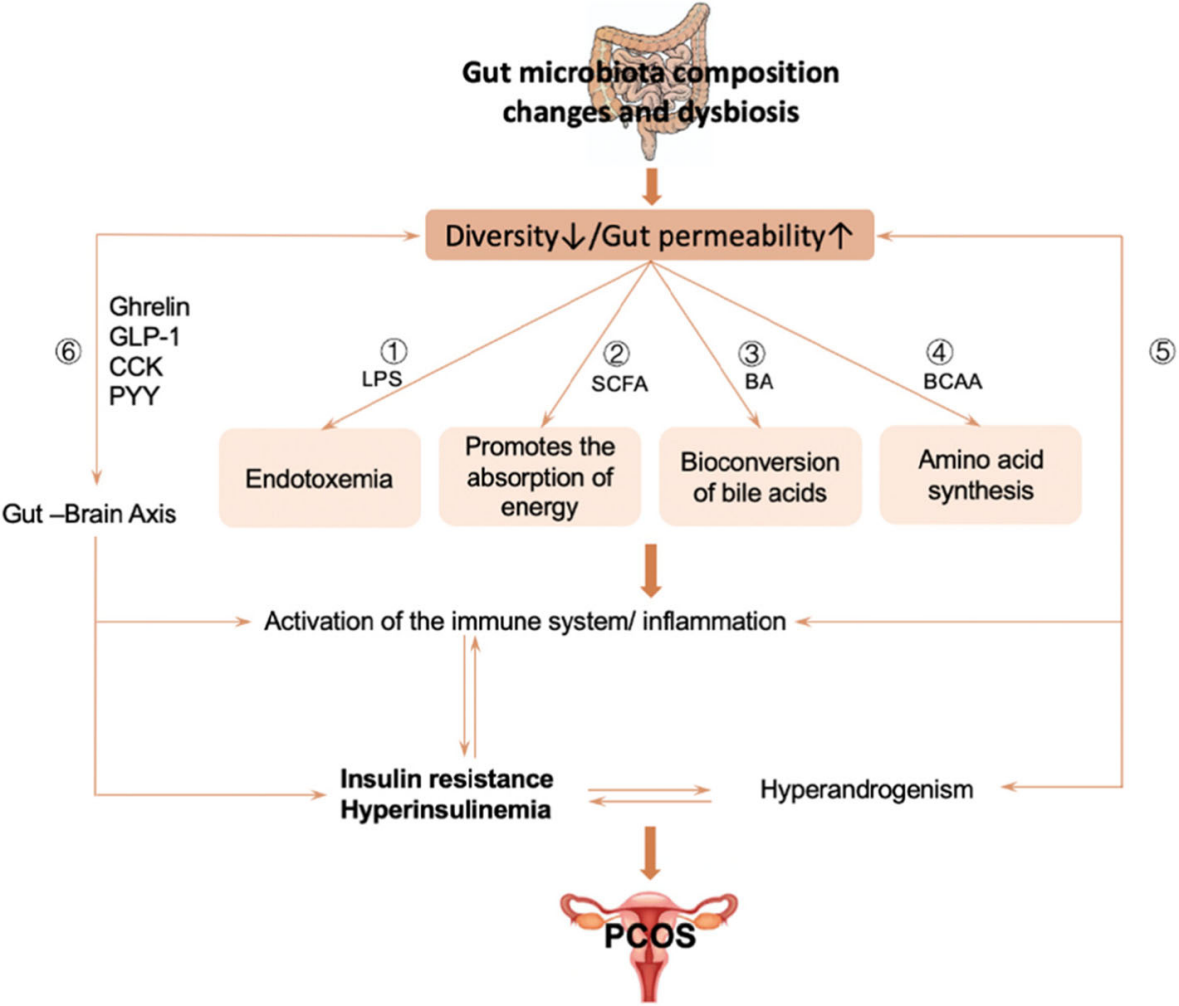
①Gut microbiota can cause IR by affecting lipopolysaccharide (LPS) and its receptor CD14(LPS-CD14; ②SCFAs protect intestinal barrier integrity and act on beta cells to promote insulin secretion, thus improving metabolism; ③Bile acids (BAs) are signaling molecules that regulate glucose metabolism and promote insulin sensitivity; ④The metabolic disorder of amino acids might aggravate IR by changing glucose metabolism or inducing inflammation;⑤A vicious cycle between hyperandrogenemia and IR in PCOS, thus promoting the occurrence and development of PCOS; ⑥Gut microbiota and its metabolites cause insulin resistance and hyperinsulinemia by stimulating the secretion of gut-brain peptides and regulating inflammation pathway activation. LPS: lipopolysaccharide; SCFA: short-chain fatty acid; BCAA: branch-chain amino acid; BA: Bile acids

Gut microbiota can be formed by LPS, BCAA (branch-chain Amino acid), SCFA(short-chain fatty acid), bile acid and other mediated inflammatory reactions affecting the sensitivity of insulin. Also, gut microbiota are associated with hyperandrogenism and gut-brain axis in women with PCOS, IR still plays an important role in them. Notably, there are a vicious cycle between hyperandrogenemia and IR in PCOS, thus promoting the occurrence and development of PCOS (Fig. 2).

## The treatment approach in PCOS

Given that the vital role of intestinal flora is regulating human metabolism and energy storage, much focus has been directed to intestinal bacteria as a new target for the treatment of obesity and related metabolic diseases. Lifestyle change involving exercise is the first step in the treatment of PCOS. Additionally, changes in diet can rapidly change the relative abundance of species making up the intestinal flora. Actually, a low-carbohydrate diet will help increase production of short-chain fatty acids which reduces incidence of chronic inflammation [93]. Moreover, high-sugar foods may be one of the inducers of PCOS, by causing intestinal flora imbalance and triggering chronic inflammation, insulin resistance, and production of androgen [17]. Notably, the relationship between exercise and intestinal flora has also been widely studied in recent years. First, Clarke et al. compared the intestinal flora structure of athletes with that of the general population and found that exercise can help in increasing the diversity of intestinal flora [94]. Clinically, insulin sensitizers (such as metformin) can improve the sensitivity of insulin receptors, endometrial receptivity, and embryo implantation and development [95]. Therefore, intestinal flora plays a vital role in the hypoglycemic mechanism of metformin. Jiang Changtao’s team found that the intestinal flora-bile acid-intestinal axis improves metabolic disorders [60]. A recent study revealed that intestinal microorganisms and their metabolites regulate PCOS-related ovarian dysfunction and insulin resistance [53]. Transplantation of *Bacteroides vulgatus* infected fecal microbes in mice resulted in ovarian dysfunction, insulin resistance, changes in bile acid metabolism, decreased secretion of interleukin-22, and infertility. Accumulating evidence has recommended probiotics, prebiotics, synbiotics as effective treatment options for PCOS patients. Ahmadi et al. showed that probiotics supplementation could reduce fasting blood glucose, serum insulin, HOMA-IR, triglyceride, and cholesterol in PCOS patients [96]. In a recent study involving probiotic intervention in PCOS patients, the consumption of probiotic *Bifidobacterium* Lactis V9 promoted the growth of SCFA producing microorganisms (such as *Faecalibacterium Praussnitzii*, *Butyriminas*, and *Akkermansia*). Moreover, changes in PYY and ghrelin levels caused fluctuations in sexual hormone levels secreted by the thalamus and thalamus through the gut-brain axis [52]. Guo et al. treated PCOS rats models with *Lactobacillus* and fecal microbiota transplantation (FMT) from healthy rats. They observed an improvement in the estrous cycles in all eight rats in the FMT group. Also, ovarian morphologies in six of the eight rats in the *Lactobacillus* transplantation group with decreased androgen biosynthesis were normalized. The composition of the gut microbiota was restored in both FMT and *Lactobacillus* treated groups. The new composition of the gut microbiota had an increased abundance of *Lactobacillus* and *Clostridium* and a decreased abundance of Prevotella [7]. Considering these results, FMT may become a new direction in the treatment of PCOS.

## Outstanding questions and recommendations

In recent years, research on intestinal microecology has attracted a lot of focus. Actually, more and more studies have confirmed that intestinal flora can regulate the synthesis and secretion of insulin, and affect androgen metabolism and follicular development. These findings have enhanced our understanding, on the etiological mechanism of PCOS. However, conflicting results on the composition and function of the gut microbiome changes in women with PCOS have only brought confusion. Nevertheless, a small sample size and regional differences have hindered current studies which aim at finding the relationship between gut microbiota and PCOS. Therefore, it is of great importance to increase the sample size and conduct a multi-point research that can supplement the existing data and establish a localized health baseline and disease model. In addition to 16S rRNA sequencing, metagenomics and metabolomics could provide exciting insights into the relationship between the gut microbiome and the occurrence of PCOS. Moreover, these analyses could confirm if variation in criteria of diagnosis could affect results of the gut microbiome composition in women with PCOS. Precisely, it is not known how changes in the gut microbiota occur in different PCOS phenotypes; therefore, there is need for further studies. Actually, present studies have partially elucidated the mechanism of intestinal flora in PCOS; however, these findings lack a comprehensive understanding of the process. Also, more randomized and controlled studies will further elucidate causes leading to intestinal microflora imbalance and its role in PCOS. The mechanism of ovulation disorder and insulin resistance in PCOS is still not precise, which has limited the development of therapeutic drugs. For instance, metformin can relieve symptoms of PCOS by positively influencing the composition and metabolites of intestinal microbiota. Zhang et al. compared the effects of Diane-35, probiotic, and berberine in the treatment of PCOS using dihydrotestosterone-induced PCOS rats [97]. Diane-35 and probiotics restored the diversity of the gut microbiota and led the recovery of gut microbiota disorders, thus improving the reproductive function in PCOS-like rats. Of note, several similar studies have confirmed the effectiveness of these treatments in improving disorders of intestinal flora from patients with in PCOS. However, their mechanisms of action are still unclear. Therefore, there is a need to understand how drugs (metformin et al.) work so as to optimize their use in the treatment of PCOS.

## Conclusion

Despite being an exogenous genetic material, intestinal flora regulates expression of host genes, leading to the occurrence of PCOS. Additionally, as a result of being a diversified ecosystem, it participates in the occurrence and development of PCOS through multiple links and pathways. Furthermore, following numerous ways and factors, it may affect the occurrence and development of IR in women with PCOS. Thus, it is imperative to further detect and analyze specific functional bacterial profiles related to the occurrence and development of PCOS. This aims at providing new targets and options for individualized. Moreover, there is need for further research to determine whether the manipulation of the intestinal microbiota can be useful in the treatment of PCOS. Lastly, it is also necessary to explore the potential use of probiotics and fecal transplant therapies in the treatment of this condition.

## Abbreviations

PCOS: Polycystic ovary syndrome
IR: Insulin resistance
T2DM: Type 2 diabetes mellitus
DOGMA: Dysbiosis of gut microbiota
LH: Luteinizing hormone
SHBG: Sex binding globulin
T: Testosterone
IGF-1: Insulin-like growth factor-1
GLUT-4: Glucose transporter 4
LPS: Lipopolysaccharide
BCAA: Branch-chain amino acid
TLR4: Toll-like receptors 4
OTUs: Operational taxonomic units
MetS: Metabolic syndrome
SCFAs: Short-chain free fatty acids
LBP: LPS binding protein
TNF-α: Tumor necrosis factor-alpha
IL-6: Interleukin6
PYY: Peptide YY
GPR41: G-protein-coupled receptor 41
FXR: Farnesoid X receptor
GPBAR1: G protein-coupled bile acid receptor 1
GUDCA: Glycoursodeoxy cholic acid
BCAAs: Branched-chain amino acids
IRS: Insulin receptor substrate
Ghrelin: Growth hormone-releasing peptide
GLP-1: Glucagon-like peptide-1
CCK: Cholecystokinin
BMI: Body mass index
FMT: Fecal microbiota transplantation
